# Optimization of 5-CQA Extraction Conditions from Green Coffee By-Product (*Coffea arabica*) Using a Response-Surface Design and the Study of Its Extraction Kinetics

**DOI:** 10.3390/molecules27175704

**Published:** 2022-09-04

**Authors:** Miriam Granados-Vallejo, Enrique Arriola-Guevara, Rosa Isela Corona-González, David Antonio Flores-Méndez, José Daniel Padilla-de la Rosa, Hugo Esquivel-Solis, Carlos Pelayo-Ortiz, Guadalupe María Guatemala-Morales

**Affiliations:** 1 Tecnología Alimentaria, Biotecnología Médica y Farmacéutica, Centro de Investigación y Asistencia en Tecnología y Diseño del Estado de Jalisco, A.C., Normalistas 800, Guadalajara C.P. 44270, Mexico; 2 Departamento de Ingeniería Química, Centro Universitario de Ciencias Exactas e Ingenierías, Universidad de Guadalajara. Blvd. Marcelino García Barragán #1421, Esq. Calzada Olímpica, Guadalajara C.P. 44430, Mexico; 3 Tecnología Alimentaria, Centro de Investigación y Asistencia en Tecnología y Diseño del Estado de Jalisco, A.C., Camino Arenero 1227, El Bajío, Zapopan C.P. 45019, Mexico; 4 Departamento de Ciencias de la Tierra y de la Vida, Centro Universitario de los Lagos, Universidad de Guadalajara, Enrique Díaz de León #1144, Lagos de Moreno C.P. 47460, Mexico

**Keywords:** green-coffee by-product, 5-CQA, extraction, optimization, kinetic

## Abstract

To take advantage of the residues generated in the production of products from green coffee and due to the special interest in the compounds contained in the bean, a by-product obtained after the extraction of the oil was studied. The physical characterization of the green-coffee-bean by-product was carried out. Subsequently, the extraction of compound 5-CQA was carried out via leaching using central composition design 2^4^ and evaluating factors such as temperature, time, solid/solvent ratio, and ethanol percentage, and its yield was quantified using HPLC. In addition, the response-surface methodology was used to maximize the efficiency of 5-CQA extraction and to perform the kinetic study. Yields of 59 ± 2 mg of 5-CQA/g from the by-product were obtained, and by selecting the best leaching conditions, the kinetic study was performed at 45, 60, and 75 °C, increasing the yield to a total of 61.8 ± 3 mg of 5-CQA/g. By applying the kinetic model of mass transfer, a fit of R^2^ > 0.97 was obtained, with $K_{L}a$ values between 0.266 and 0.320 min^−1^. This study showed an approach to optimize the 5-CQA extraction conditions, resulting in a simple, fast, reproducible, accurate, and low-cost method.

## 1. Introduction

Coffee is one of the most consumed beverages in the world, prepared from roasted coffee beans, which are considered the second most valuable export product and the most traded and commercialized after petroleum [1,2]. Globally, about 10.47 million tons of coffee are produced per year in more than 50 countries [3]. Among the bioactive compounds in coffee, polyphenols stand out for their antioxidant properties, which have metabolic benefits [4]. Within coffee, there is chlorogenic acid (CGA), which is one of the most abundant polyphenols in green coffee beans [5]. Structurally, CGA is a family of esters formed between caffeic acid and quinic acids, and 5-O-caffeoylquinic acid (5-CQA) is the most common isomer in CGA. Recently, 5-CQA has gained attention due to several important and therapeutic functions, such as antioxidant, antibacterial, antihypertensive, hepatoprotective, cardioprotective, antipyretic, antiviral, and antiobesity functions, among others [6]. Furthermore, as the global economy mends, the 2021 growth of chlorogenic acid had a significant change from the previous year. According to a study conducted by Market and Research, the market size for this compound will reach 175.8 USD million in 2028, growing at a CAGR of 3.2% during 2022–2028 [7]. However, the coffee industry is in crisis due to oversupply over demand causing a decrease in coffee price; therefore, the development of value-added products from coffee or coffee industry waste could be a solution to stop this trend [8]. 

Currently, another commercial activity has been introduced in the coffee industry, the extraction of oil from green coffee bean by means of pressing, which to some extent generates a by-product known as pressed cake. This by-product still contains a substantial content of oil and also presents a great content of bioactive compounds, which are mainly hydrophilic, that remain in the biomass and that could be of interest in different sectors [9]. In recent years, the use of coffee by-products has been suggested as a promising source of functional and bioactive chemicals for the food, pharmaceutical, and cosmetic industries. However, the obtained by-product known as pressed cake is a chemically rich material that is usually neglected by the coffee industry, and there is not enough information in the scientific literature concerning its chemical composition and biological properties. Thus, this is an economically valuable industrial output that can be exploited [10].

Generally, there are a variety of methods for extraction, including solid–liquid extraction with organic solvents, ultrasound-assisted extraction, microwave-assisted extraction, supercritical fluid extraction, and high-pressure processes [11]. However, in order to exploit this by-product, solid–liquid extraction may be an interesting proposal as a first step [9,12]. In particular, for the extraction processes of phenolic compounds and chlorogenic acids, in order to achieve the highest recovery of said compounds, we studied how certain variables that affect the process are the type of solvent and concentration, the ratio of grams of sample per volume of solvent, the number of extraction stages, pH, extraction time, temperature, particle size of the solid matrix, and agitation [13,14]. In this sense, the response-surface methodology (RSM) is commonly used to achieve optimized extraction conditions, and it is important because it allows a more adequate and complete exploitation of the raw material to be conducted, evaluates the relative importance of each independent variable, and determines the optimal operating conditions for the predicted responses [15,16]. In addition, it has been successfully applied to optimize parameters in various investigations, as performed by Pavlovi’c et al. (2013) and Pettinato et al. (2019), for instance, the recovery of antioxidants from spent filter coffee [17,18].

Therefore, the objective of this study was the valorization of a green-coffee by-product (pressed cake), which was used as raw material for the extraction of 5-CQA. For this purpose, the extraction conditions (temperature, time, solid/solvent ratio, and ethanol percentage) were optimized by means of leaching using the response-surface methodology. In addition, based on the optimal conditions, a mathematical model was proposed to describe the extraction kinetics.

## 2. Results and Discussion

### 2.1. Characterization of Raw Material

**Figure 1:**
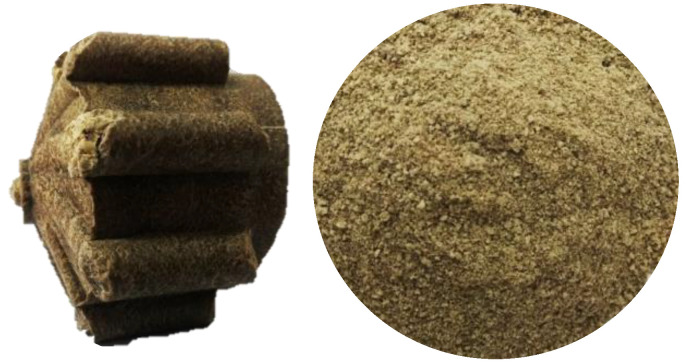
Green-coffee by-product before and after grinding.

From a green-coffee by-product, we obtained a moisture content of 7%, a value that is ideal to avoid fungal growth and is within the permitted range according to NMX-F-177-SCFI-2009 [19]; this value was also used to express the results of 5-CQA quantification on a dry-weight basis. The particle size distribution was 0.44 mm (Figure 1) and coincided with the size range suggested for the industrial production of extracts [20]. This parameter is important because it is directly related to the internal mass strength. Therefore, to increase the overall extraction yield, a reduction in the particle size increases the contact surface between the solid and the solvent, thus decreasing the distance the solute travels within the porous particle to the surface [21]. This effect was demonstrated by several authors, with the extraction yield being considerably improved, as reported by Jokić et al. (2012) [22] in his work to improve the yield of soybean oil, by Oliveira et al. (2018) [23] in obtaining an extract rich in diterpenes from green coffee, and by Gião et al. (2009) [24] in obtaining an extract with antioxidant properties from a vegetable source. Consequently, the particle size of plant matrices is an important consideration in extraction.

### 2.2. 5-CQA Quantification via Liquid Chromatography

The yields obtained in the extracts were 15–59 mg 5-CQA/g of dry green coffee (Table 1). The average maximum yield obtained in one of the extracts was 59.62 ± 2.36 mg/g, a value that was higher than those already reported by other authors. In a study where researchers worked with the extraction conditions to obtain a product rich in antioxidants from green coffee beans, values of 30.19 ± 0.53 mg/g of dry sample were obtained, using as solvent an isopropanol–water solution; later, optimization was performed, and the maximum value was 56 ± 11 mg/g of dry sample [8]. In other studies, to obtain chlorogenic acids, spent coffee and roasted coffee were used, and the concentration obtained was 54.6 ± 0.7 mg CGA/g of total solids [25] and 32.3 mg CGA/g of dry sample [26], respectively. Finally, in an investigation carried out for the extraction of CGA from green coffee, the highest value obtained was 176.7 ± 12.0 mg CGA/g of dry sample [27], so this last value is higher than that reported in the present work.

### 2.3. Optimization of 5-CQA Extraction Parameters from Green-Coffee By-Product via RSM

The experimental design for 5-CQA was based on a four-factor two-level central composition design (CCD). Table 1 shows the CCD with 28 experimental runs and the corresponding extraction yield response. The RSM method was used to investigate the interactions between the factors in the extraction and optimize the independent variables. The following quadratic equation was obtained using multiple regression analysis based on the experimental data:

(1) $$Y=45.6171-0.2698X_{1}+0.3328X_{2}-0.1985X_{3}+0.1783X_{4}+0.0057X_{1}X_{2}\;-0.0025X_{1}X_{3}+0.0029X_{1}X_{4}-0.0033X_{2}X_{3}+0.0024X_{2}X_{4}+0.0029X_{3}X_{4}+0.0034X_{1}^{2}-0.0078X_{2}^{2}+0.0003X_{3}^{2}-0.0052X_{4}^{2}$$

where Y is the 5-CQA extraction yield (mg/g) and $X_{1}$, $X_{2}$, $X_{3}$, and $X_{4}$ are variables coding temperature (°C), time (min), solid/solvent ratio (mg/mL), and ethanol (%v/v), respectively. The equation expressed can be used to predict the response of each factor at a given level, and the relative impact of these factors can be identified by comparing the coefficients of the factors. Statistical tests in the form of an analysis of variance (ANOVA) were performed to study the goodness-of-fit and the appropriateness of the model to the responses in the fitted quadratic polynomial model of 5-CQA extraction yields.

#### 2.3.1. Statistical Analysis of the Model

The results of the ANOVA for the extraction yields of 5-CQA are presented in Table 2. The model’s F-value of 13.43 implies the model was significant; this was corroborated by the value of *p* < 0.0001, indicating that there was only a probability of 0.01% that a higher F-value could occur due to noise. *p*-values less than 0.05 indicated that the model terms were significant, and in this model, X_1_, X_3_, X_4_, X_3_X_4_, and X_4_^2^ were significant model terms. A significant lack of fit (*p* < 0.05) indicates the failure of the model to represent the data in the experimental domain, and these points are not to be included in the regression [28]. In the present investigation, the F-value and *p*-value of the lack of fit were 0.9433 and 0.5912, respectively, indicating that the model equation was suitable for predicting the extraction yield of 5-CQA.

The coefficient of determination (R^2^) was 0.9353 for the extraction yield of 5-CQA, inferring that the accuracy and general predictive ability of the quadratic polynomial regression model represented by Equation (1) was very good, since, for a good fit of the model, R^2^ should not be less than 80% [29,30]. Furthermore, the discrepancy between the predicted R^2^ value and the adjusted R^2^ value was less than 0.2, indicating that they were in the reasonable range of fluctuation (Table 3). 

The coefficient of variation (C.V.) is the ratio of the standard deviation to the mean and shows the extent of the variability in the mean. Small values of the C.V. represent better reproducibility. Overall, a C.V. higher than 10% signifies that the variation in the mean value is high and does not satisfactorily develop an adequate response model [31]. In this study, a value of 8.75% demonstrated that the model adequately explained the response. Adequacy accuracy is a signal-to-noise ratio. It compares the range of predicted values at the design points to the mean prediction error, and a value greater than 4 is adequate [29,32], which was verified in our case (15.5083). Thus, the regression equation could be applied for the prediction of the optimal conditions of the extraction process.

#### 2.3.2. Analysis of Response Surface

**Figure 2:**
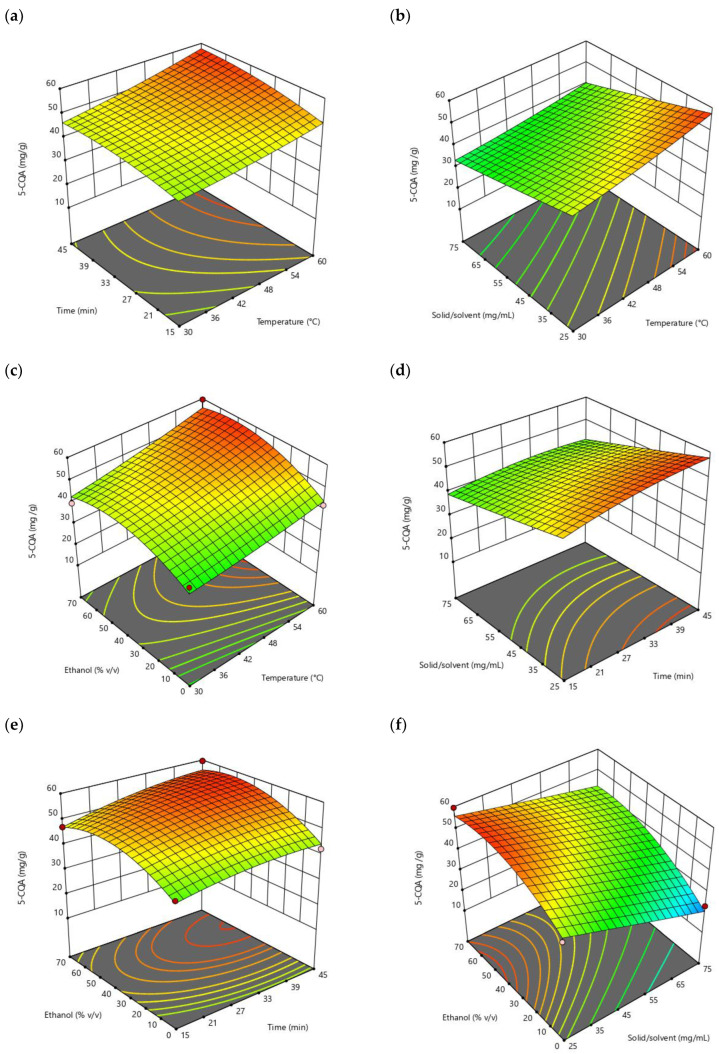
Response-surface representations for the yield of 5-CQA: (**a**) varying temperature and time; (**b**) varying temperature and solid/solvent ratio; (**c**) varying temperature and % ethanol; (**d**) varying time and solid/solvent ratio; (**e**) varying time and % ethanol; (**f**) varying solid/solvent ratio and % ethanol.

The response surfaces can be illustrated as three-dimensional plots as presented in Figure 2, which shows the response surface for the 5-CQA yield as a function of two variables. In each of the interactions shown in the different graphs, it was observed how temperature (X_1_), time (X_2_), solid/solvent ratio (X_3_), and ethanol percentage (X_4_) influenced the extraction yield of 5-CQA. In Figure 2a, the maximum yield values were found in the combination of the highest temperature and time; for this particular case, only the temperature variable was significant in the design.

In the interaction between factors X_1_ and X_3_, it was observed that increasing the temperature and having a low solid/solvent ratio increased the yield of 5-CQA (Figure 2b). In Figure 2c, the region that had the highest yields was found by combining a high temperature and an ethanol percentage between 40 and 60%. In the relationship between factors X_2_ and X_3_ shown in Figure 2d, it could be observed that the solvent/solvent ratio at its lowest levels has a marked zone for obtaining maximum yields, while time had a wide interval as it was not a significant factor; the same occurred as shown in Figure 2e, in which the interaction of variables X_2_ and X_4_ showed a poorly defined zone for obtaining maximum yields. Finally, the synergistic effects of X_3_ with X_4_ were the most significant in improving the yield of 5-CQA, as the *p*-values were less than 0.05. It could be observed that the region where the highest 5-CQA concentrations were reached was when ethanol percentages were at their mid-high levels (40–60%) in combination with a low solid/solvent ratio (Figure 2f).

What was obtained in the statistical analysis for the yield of 5-CQA agrees with what was mentioned by authors such as Soto-García and Rosales-Castro, (2016) [33], who reported that the concentrations in plant extracts with antioxidant compounds are directly affected by the mass–solvent ratio, as well as the percentage of solvent used. In addition, Sharapin et al. (2000) [20] mentioned that factors such as the percentage in which a solvent is used selectively determine or not the extraction of certain compounds, so it can become one of the most influential factors in the process.

#### 2.3.3. Optimization of Extraction Parameters and Verification of Optimized Conditions

To determine the optimal operating conditions, the fitted model was used to predict the yield of 5-CQA, which was set to a maximum, and the extraction parameters were set to the appropriate range. Numerical optimization was selected because it locates a point that maximizes the desirability function [34]. The predicted optimum conditions for 5-CQA extraction were calculated as a temperature of 60 °C, a time interval of 45 min, a solid/solvent ratio of 25 mg/mL, and ethanol at 51.09% (taking into account actual operating conditions, the percentage of ethanol was modified to 50%), and the model predicted a maximum 5-CQA yield of 57.76 mg/g. To verify the optimum conditions predicted using RSM, extraction experiments were conducted in triplicate under the optimum conditions. It was demonstrated that the experimental average extraction yield was 55.47 ± 2.68 mg/g, similar to the predicted value (57.76), indicating that the established model was statistically reliable.

According to everything previously analyzed, it could be determined that the extract obtained from the green-coffee by-product, which are currently discarded, could be considered a natural source with a high content of 5-CQA. This extract could be used in dietary supplements, cosmetics, or pharmaceutical formulations as an antioxidant agent or as a source of important coffee secondary metabolites. Furthermore, this research study has also importance from an environmental point of view and can meet the principles of green chemistry through the reuse of by-products generated by the industry, minimizing their disposal in the environment.

### 2.4. Analysis of 5-CQA Extraction Kinetics 

In recent years, the solid–liquid extraction of bioactive compounds from natural sources has received considerable attention, while kinetic modeling is a valuable tool for understanding the complex heating and mass transfer behavior during extraction [1,35]. Therefore, the extraction kinetics of 5-CQA were studied, as was the influence of the temperature over the volumetric mass transfer coefficient. 

**Figure 3:**
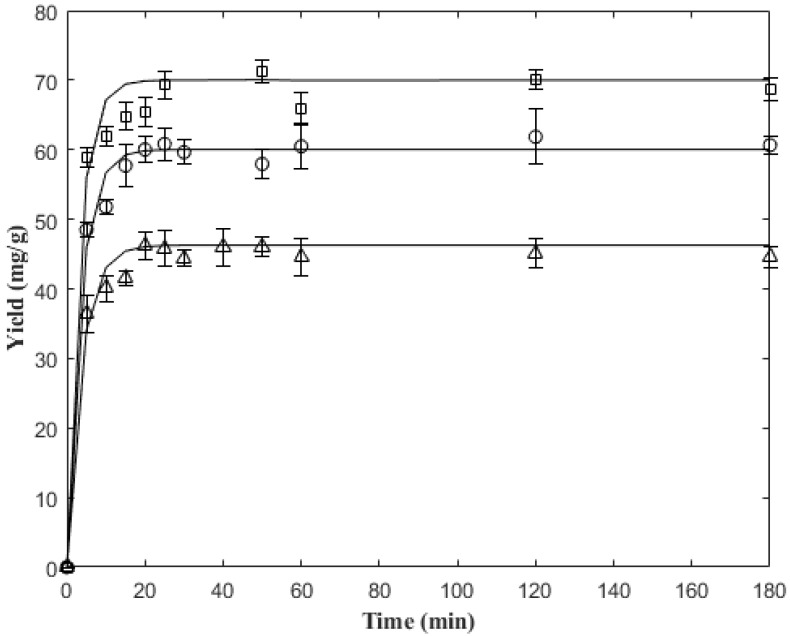
Extraction kinetics at different temperatures: model prediction versus experimental data at 45 °C (Δ), 60 °C (o), and 75 °C (□).

Figure 3 shows the comparison between the predictions of the proposed model against the experimental data of 5-CQA extraction at different temperatures (45, 60, and 75 °C); it was seen that the model and estimated $K_{L}a$ adequately described the extraction kinetics, reaching fits higher than 97%, as shown in Table 4 (R^2^ > 0.97). As a precedent, the same mass transfer kinetic model was employed by Handayani et al. (2008) [36] and Lau et al. (2015) [37], who reported coefficients of determination of 0.98 and 0.97 for the extraction of astaxanthin and rosmarinic acid, respectively. These coefficients were similar to those obtained in this work.

The extraction kinetics showed that from minute 25, the asymptotic region was reached, recovering between 97–99% of 5-CQA during this time. A pattern that was observed in other studies of the same type shows that the increase in temperature increases the diffusivity coefficient and the mass transfer rate [36,38,39]. In this study, a 53.8% increase in the yield of 5-CQA was observed when the temperature was increased from 45 °C to 75 °C. This increase indicates that 5-CQA is a thermo-resistant phenolic compound and could be extracted at a higher temperature range [40]. However, it must be taken into account that some biologically active important substances can be degraded at high temperatures [39].

**Figure 4:**
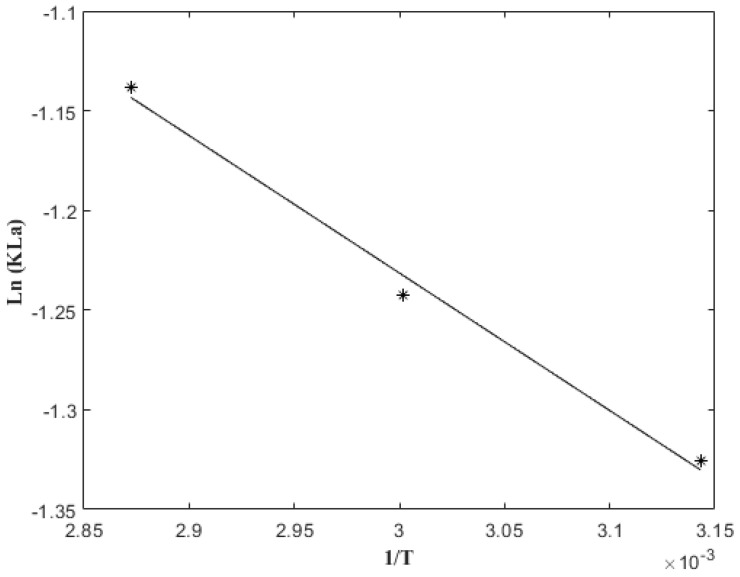
Activation energy calculation from plot of Ln $K_{L}a$ versus 1/T (*).

$K_{L}a$ values also increased with the temperature, as shown in Table 2. The dependence of $K_{L}a$ on temperature was established with the Arrhenius equation, which was written in a linearized form, plotting Ln ($K_{L}a$) versus 1/T, as shown in Figure 4. From the slope and the intercept, the activation energy ($E_{a}$ = 5.73 kJ/mol) and the frequency factor (A = 2.31 min^−1^) were obtained. The value obtained for the activation energy in the present investigation was similar to the 9.74 kJ/mol reported by Dibert and Andrieu, (1989) [41] for the extraction of chlorogenic acid with green coffee. Some reports in the literature mentioned that when the activation energy is less than 20 kJ/mol, the extraction process from solid to liquid is controlled by diffusion; such was the case of 5-CQA in this study.

The purpose of the extraction model, as well as $K_{L}a$ estimation, was to provide us with enough information to scale batch extraction and even design an extraction process in a continuous system. 

### 2.5. Extraction by Stages

**Figure 5:**
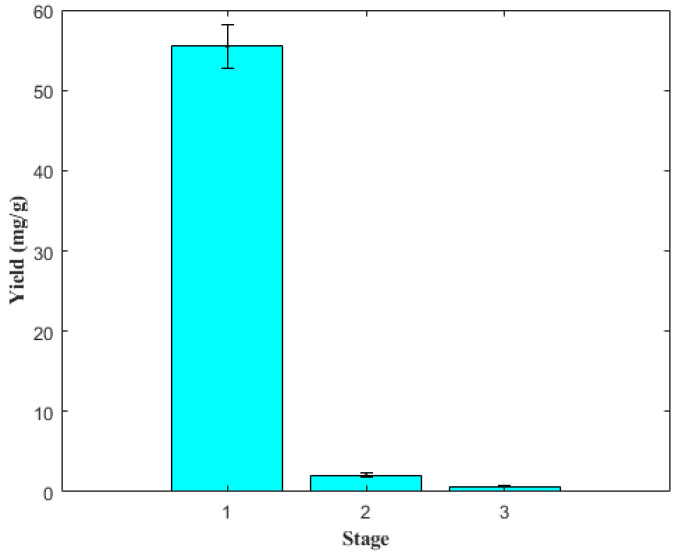
Yields of 5-CQA per extraction stage.

To evaluate whether it is possible to increase the extraction yield of 5-CQA, the process was performed in three stages. Figure 5 shows the yield of 5-CQA by stages; in the first extraction, the yield achieved (55.47 ± 2.68 mg/g) was considerably higher than those of stage 2 (2.0 ± 0.26 mg/g) and stage 3 (0.72 ± 0.04 mg/g). Thus, the recovery of 5-CQA in the first stage was 95.3%; in the second, 3.4%; and in the third, 1.2%, respectively. The results presented for the extraction stages were similar to those reported by Butiuk et al. (2021) [39] and Liu et al. (2010) [42], who recovered more than 90% of chlorogenic acid in the first extraction stage. 

The overall yield of 5-CQA achieved in the three stages was 58.19 mg/g. This value differs from that obtained using the kinetics at 60 °C (61.85 mg/g; Table 4). However, it should be noted that increasing the extraction time by incorporating more stages and adding fresh solvent could influence the oxidation of polyphenolic compounds, reducing the extraction efficiency [43,44]. 

## 3. Materials and Methods

### 3.1. Raw Material

A by-product known as pressed cake, generated from the process of defatting green coffee beans (*Coffea arabica*) by means of cold pressing, was used. Green coffee beans were from Talpa de Allende, Jalisco, Mexico. Defatted green-coffee by-product in the form of cake was processed in a disk mill (TA PPA-44; MAREN, CDMX, Mexico) for size reduction. Subsequently, it was characterized by determining the moisture content using the NMX-F-083-1986 standard [45] and the particle size distribution (PSD) using a sieve (RX-29; WS Tyler, Mentor, OH, USA); the latter was calculated using Equation (2):

(2) $$PSD=\frac{W_{1}D_{1}+W_{2}D_{2}+\ldots +W_{n}D_{n}}{WT}$$

where $W_{1-n}$ is the weight of particles retained on each sieve, $D_{1-n}$ is the mesh aperture of each sieve, and WT is the total weight of the processed sample.

### 3.2. Reagents

Phosphoric acid (H_3_PO_4_) was purchased from Karal (Leon, Gto, Mexico); methanol (CH_3_OH) was purchased Sigma-Aldrich Co. (St. Louis, MO, USA); and ethanol (C_2_H_5_OH) was of reactive grade (Zapopan, Jal, Mexico).

### 3.3. Extraction of 5-CQA from Green-Coffee By-Product

The extracts were prepared via leaching according to the method by Meinhart et al. (2017) [46] and Ruiz-Palomino et al. (2019) [47] with modifications. In a 100 mL volumetric flask, the previously weighed quantity (in grams) of green-coffee by-product was added, and 25 mL of the solvent was added at different concentrations for each experiment. An isothermal heating system was set up using a beaker with demineralized water to ensure convective heat transfer to the flask. The design temperature was set, and the flask was shaken at 120 pm for the indicated time intervals using a digital shaking plate (C-MAG HS4; IKA Words Inc., Staufen, Brisgovia, Alemania); at the end of extraction, the flask was gauged to 100 mL with the corresponding solution according to the design. Subsequently, filtration was performed to separate the supernatant, using Whatman No. 1 filter paper to retain the solids; finally, the extracts were stored in amber bottles and refrigerated at 4 °C until analyses.

### 3.4. HPLC Analysis of Extracts

The extracts from green-coffee by-product (EGCP) were analyzed using high-performance liquid chromatography (HPLC). A chromatograph with a 996-diode array detector (Waters Corporation, Milford, MA, USA) was used. A volume of 20 µL of the extracts was injected onto a Kromasil C18 5 µm column (4.6 mm × 250 mm). Mobile phase A was 5 mM phosphoric acid solution, and mobile phase B consisted of HPLC-grade methanol. The gradient mode was initially set at an A/B ratio of 85/15 from 0 to 5 min, 80/20 from 6 to 10 min, 60/40 from 11 to 20 min, 70/30 from 21 to 25 min, 80/20 from 26 to 30 min, and finally 80/20 from 31 to 35 min. The feed flow rate was 1 mL/min, and the analysis was performed at room temperature. The UV detector was set at a wavelength of 325 nm to measure the absorbance of 5-CQA.

### 3.5. Extraction Optimization via RSM

To further evaluate the main factors affecting extraction, the operating conditions were optimized using RSM and central composite design 2^4^, which includes center points and star points centered on the faces. The variables and levels studied were the temperature (30 to 60 °C), time (15 to 45 min), the solid/solvent ratio (25 to 75 mg/mL), and the percentage of ethanol in the extraction solution (0 to 70%). The 5-CQA yield was set as the response.

According to the experimental design, extraction was performed under different conditions, and 28 experiments were carried out. The model was fitted with the quadratic equation given below:

(3) $$Y=b_{0}+\overset{4}{\underset{i=1}{\sum }}b_{i}X_{i}+\overset{4}{\underset{i=1}{\sum }}b_{ii}X_{i}^{2}+\overset{4}{\underset{i=1}{\sum }}\overset{4}{\underset{j=i+1}{\sum }}b_{ij}X_{i}X_{j}$$

where Y represents the response variable; $b_{0}$ is the constant term of the regression equation; coefficients $b_{i}$ are the linear terms, $b_{ii}$ are the quadratic terms, while $b_{ij}$ is the interaction terms; and $X_{i}$ and $X_{j}$ are the independent encoded variables. 

Data analysis, response surfaces, polynomial models, and ANOVA were performed with Design-Expert 11 software (Stat-Ease Inc., Minneapolis, MN, USA). The statistical significance of the variables was determined at a probability level of 5% (*p* < 0.05).

### 3.6. Kinetic Model

**Figure 6:**
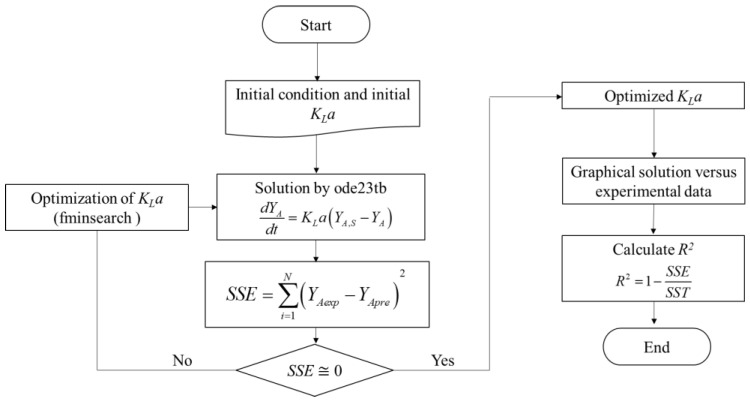
Optimization algorithm for the solution of the differential equation and the estimation of $K_{L}a$.

According to Geankoplis (1993) [48], when a material dissolves from solid form in a solvent solution, the rate of mass transfer from the solid surface to the liquid is usually the controlling factor. For a batch system, from the mass balance, the rate of transfer of 5-CQA from solid form (by-product) in bulk liquid (solvent) can be written as:

(4) $$\frac{dN_{A}}{dt}=K_{L}A\left(C_{A,S}-C_{A}\right)$$

where $K_{L}$ is the mass transfer coefficient in the liquid phase, A is the surface area of the particles, and $C_{A,S}$ and $C_{A}$ are the concentrations of 5-CQA on the surface of the solid and in the bulk liquid at time (t), respectively. Assuming that extraction is performed at constant volume, the accumulation term can be rewritten as a function of the 5-CQA concentration:

(5) $$\frac{dC_{A}}{dt}=K_{L}a\left(C_{A,S}-C_{A}\right)$$

where a is the interfacial surface (A/V), while term $K_{L}a$ is defined as the volumetric mass transfer coefficient. At the beginning of extraction (t = 0), it is considered that the concentration of 5-CQA in the liquid is zero ($C_{A}$ = 0). Equation (5) can be written in terms of the yield ($Y_{A}$), i.e., in milligrams of 5-CQA per gram of dry sample:

(6) $$\frac{dY_{A}}{dt}=K_{L}a\left(Y_{A,S}-Y_{A}\right)$$

where $Y_{A}$ and $Y_{A,S}$ are the 5-CQA yields in the bulk liquid and at equilibrium per mass of by-product, respectively. The solution of Equation (6) and the estimation of $K_{L}a$ were performed simultaneously in MATLAB numerically using functions ode23tb and fminsearch as shown in the optimization algorithm described in Figure 6, where the objective function was to minimize the sum of the squared error (*SSE*) between the experimental data ($Y_{Aexp}$) and the data predicted by the model ($Y_{Apre}$). The coefficient of determination, R^2^, was evaluated to determine the fit of the model to the experimental data.

To establish the effect of the temperature on the extraction of 5-CQA, the analysis of the extraction kinetics was performed under the optimal conditions of the experimental design using different temperatures (30, 60, and 75 °C). Then, $K_{L}a$ was written using the Arrhenius equation in a linearized form:

(7) $$\mathrm{Ln}\;K_{L}a=\mathrm{Ln}A-\frac{E_{a}}{RT}$$

where $E_{a}$ is the activation energy, R is the universal gas constant (8.31 J/mol K), A is the pre-exponential factor or frequency factor (min^−1^), and T is the absolute temperature (K).

### 3.7. Effect of Extraction in Stages

Three successive extractions were performed under the optimal conditions of Section 3.5 according to the method described in Section 3.3. After the first extraction, the solid residue was resuspended in 100 mL of fresh solvent. The extraction process was repeated twice.

## 4. Conclusions

The leaching process used, although it is a conventional method, provided some advantages; for example, the traditional solvents were replaced by ethanol, reducing the potentially negative environmental impact and creating more ecological and environmentally friendly extraction techniques.

This study showed an approach to optimize the 5-CQA extraction conditions. The optimal condition consisted of a temperature of 60 °C, a time interval of 45 min, a solid/solvent ratio of 25 mg/mL, and ethanol at 50%, resulting in a simple, fast, reproducible, accurate, and low-cost method. Observing that good yields were obtained with leaching, the use of extraction methods with emerging technologies can be considered, with the aim of improving the obtaining of compounds of interest such as 5-CQA. Through the Arrhenius equation, it was possible to determine the dependence of $K_{L}a$ on the temperature and the nature of the process, which was diffusional.

Moreover, the present method can be easily used in coffee waste utilization laboratories and could also be seen as the first step in research aiming to develop new 5-CQA-rich products from this green-coffee by-product as antioxidant agents in cosmetics or pharmaceutical formulations, and dietary supplements.

## Figures and Tables

**Table 1:** Central composite design matrix and results obtained in the study of 5-CQA extraction yield from green-coffee by-product.

Run	Variables ^a^				5-CQA Extraction Yield (mg/g)	
	X_1_	X_2_	X_3_	X_4_	Experimental Data ^b^	RSM-Predicted
1	30	15	75	0	22.56 ± 0.61	23.71
2	45	30	50	35	49.49 ± 2.27	43.33
3	45	30	75	35	38.8 ± 1.68	36.47
4	60	45	75	0	23.32 ± 0.06	20.71
5	45	30	50	35	43.60 ± 0.22	43.33
6	30	45	75	0	15.11 ± 1.23	17.43
7	45	15	50	35	39.47 ± 3.60	40.92
8	45	30	25	35	46.58 ± 2.39	50.51
9	45	30	50	35	43.61 ± 1.46	43.33
10	30	15	25	70	39.04 ± 0.85	39.02
11	30	45	25	70	39.78 ± 2.40	42.76
12	60	45	25	70	59.62 ± 2.36	55.85
13	45	30	50	70	43.8 ± 1.83	42.49
14	30	30	50	35	42.28 ± 2.62	41.29
15	60	30	50	35	44.31 ± 0.51	46.90
16	45	30	50	0	28.4 ± 3.69	31.31
17	60	15	75	0	22.58 ± 0.09	21.83
18	30	45	75	70	34.94 ± 0.24	33.21
19	30	15	25	0	39.8 ± 0.06	38.52
20	30	45	25	0	39.94 ± 3.12	37.19
21	60	15	25	70	47.06 ± 0.68	46.96
22	60	45	75	70	39.07 ± 1.84	42.57
23	60	45	25	0	42.28 ± 2.47	44.19
24	45	30	50	35	41.43 ± 0.14	43.33
25	60	15	75	70	38.50 ± 1.28	38.63
26	30	15	75	70	34.11 ± 0.84	34.42
27	60	15	25	0	41.27 ± 0.44	40.37
28	45	45	50	35	42.08 ± 0.71	42.23

^a^ Independent variables: X_1_, temperature (°C); X_2_, time (min); X_3_, solid/solvent ratio (mg/mL); X_4_, ethanol (%*v/v*). ^b^ Average values of the determinations (±SDs; *n* = 2).

**Table 2:** ANOVA results from CCD for extraction yields of 5-CQA.

Source	Sum of Squares	DF	Mean Square	F-Value	*p*-Value
Model	2153.36	14	153.81	13.43	<0.0001
X_1_^a^	141.40	1	141.40	12.35	0.0038
X_2_^a^	7.67	1	7.67	0.6698	0.4279
X_3_^a^	887.33	1	887.33	77.48	<0.0001
X_4_^a^	562.91	1	562.91	49.15	<0.0001
X_1_X_2_	26.57	1	26.57	2.32	0.1516
X_1_X_3_	13.91	1	13.91	1.21	0.2904
X_1_X_4_	37.03	1	37.03	3.23	0.0954
X_2_X_3_	24.40	1	24.40	2.13	0.1681
X_2_X_4_	25.65	1	25.65	2.24	0.1583
X_3_X_4_	104.24	1	104.24	9.10	0.0099
X_1_^2^	1.53	1	1.53	0.1333	0.7209
X_2_^2^	7.91	1	7.91	0.6903	0.4211
X_3_^2^	0.0697	1	0.0697	0.0061	0.9390
X_4_^2^	106.50	1	106.50	9.30	0.0093
Residual	148.88	13	11.45		
Lack of Fit	112.95	10	11.30	0.9433	0.5912
Pure Error	35.92	3	11.97		
Cor Total	2302.24	27			

^a^ Independent variables: X_1_, temperature (°C); X_2_, time (min); X_3_, solid/solvent ratio (mg/mL); X_4_, ethanol (%*v/v*). DF: Degree of freedom; F-Value: Fisher distribution value; *p*-Value: Significance.

**Table 3:** Analysis of variance for the fitted models (Fit statistics).

Fit Statistics	Yield (mg/g)
R^2^	0.9353
Adjusted R^2^	0.8657
Predicted R^2^	0.6690
C.V. %	8.75
Adeq Precision	15.5083

**Table 4:** Estimated $K_{L}a$ values, equilibrium yields, and correlation coefficients at different temperatures.

T (°C)	T (K)	$K_{L}a\;(\min^{-1})$	$Y_{A,S}\;(\mathrm{mg}/g)$	R^2^
45	318.15	0.266	46.0356	0.978
60	333.15	0.289	61.8535	0.987
75	348.15	0.320	71.1760	0.976

## Data Availability

Not applicable.

## Sample Availability

Samples of green-coffee by-products and extracts are available from the authors.

## References

[1] Chen Q., Dong W., Wei C., Hu R., Long Y. (2020). Combining integrated ultrasonic-microwave technique with ethanol to maximise extraction of green coffee oil from Arabica coffee beans. Ind. Crops Prod..

[2] Bae J.H., Park J.H., Im S.S., Song D.K. (2014). Coffee and health. Integr. Med. Res..

[3] USDA Foreign Agricultural Service “Coffee: World Markets and Trade” 2022. https://www.fas.usda.gov/data/coffee-world-markets-and-trade.

[4] Wan C.W., Wong C.N.Y., Pin W.K., Wong M.H.Y., Kwok C.Y., Chan R.Y.K., Yu P.H.F., Chan S.W. (2013). Chlorogenic acid exhibits cholesterol lowering and fatty liver attenuating properties by up-regulating the gene expression of PPAR-α in hypercholesterolemic rats induced with a high-cholesterol diet. Phyther. Res..

[5] Amigoni L., Stuknytė M., Ciaramelli C., Magoni C., Bruni I., De Noni I., Airoldi C., Regonesi M.E., Palmioli A. (2017). Green coffee extract enhances oxidative stress resistance and delays aging in *Caenorhabditis elegans*. J. Funct. Foods.

[6] Rofouei M.K., Kojoori S.M.H., Moazeni-Pourasil R.S. (2021). Optimization of chlorogenic acid extraction from Elm tree, *Ulmus minor* Mill., fruits, using response surface methodology. Sep. Purif. Technol..

[7] Market and Research, “Chlorogenic Acid Market”, United States, 2022. https://www.marketandresearch.biz/report/235796/global-chlorogenic-acid-market-growth-2022-2028?fbclid=IwAR0Lt5or7XbIWnxH3RA2NiK5KzpX36uuPQ--yz3Q-SvrjDbnC2yoOg2R4IM.

[8] Madhava M., Sulochanamma G., Sampathu S.R., Srinivas P. (2008). Studies on extraction and antioxidant potential of green coffee. Food Chem..

[9] Oliveira É.R., Silva R.F., Santos P.R., Queiroz F. (2019). Potential of alternative solvents to extract biologically active compounds from green coffee beans and its residue from the oil industry. Food Bioprod. Process..

[10] Castro A.C., Oda F.B., Almeida-Cincotto M.G.J., Davanço M.G., Chiari-Andréo B.G., Cicarelli R.M.B., Peccinini R.G., Zocolo G.J., Ribeiro P.R.V., Corrêa M.A. (2018). Green coffee seed residue: A sustainable source of antioxidant compounds. Food Chem..

[11] Jovanović A.A., Đorđević V.B., Zdunić G.M., Pljevljakušić D.S., Šavikin K.P., Gođevac D.M., Bugarski B.M. (2017). Optimization of the extraction process of polyphenols from *Thymus serpyllum* L. herb using maceration, heat- and ultrasound-assisted techniques. Sep. Purif. Technol..

[12] Zhu F., Du B., Zheng L., Li J. (2015). Advance on the bioactivity and potential applications of dietary fibre from grape pomace. Food Chem..

[13] Mussatto S.I., Ballesteros L.F., Martins S., Teixeira J.A. (2011). Extraction of antioxidant phenolic compounds from spent coffee grounds. Sep. Purif. Technol..

[14] Vieitez I., Maceiras L., Jachmanián I., Alborés S. (2018). Antioxidant and antibacterial activity of different extracts from herbs obtained by maceration or supercritical technology. J. Supercrit. Fluids.

[15] Ballesteros L.F., Ramirez M.J., Orrego C.E., Teixeira J.A., Mussatto S.I. (2017). Optimization of autohydrolysis conditions to extract antioxidant phenolic compounds from spent coffee grounds. J. Food Eng..

[16] Kuo C.H., Liu T.A., Chen J.H., Chang C.M.J., Shieh C.J. (2014). Response surface methodology and artificial neural network optimized synthesis of enzymatic 2-phenylethyl acetate in a solvent-free system. Biocatal. Agric. Biotechnol..

[17] Pavlović M.D., Buntić A.V., Šiler-Marinković S.S., Dimitrijević-Branković S.I. (2013). Ethanol influenced fast microwave-assisted extraction for natural antioxidants obtaining from spent filter coffee. Sep. Purif. Technol..

[18] Pettinato M., Casazza A.A., Ferrari P.F., Palombo D., Perego P. (2019). Eco-sustainable recovery of antioxidants from spent coffee grounds by microwave-assisted extraction: Process optimization, kinetic modeling and biological validation. Food Bioprod. Process..

[19] (2009). Café Verde de Especialidad—Especificaciones, Clasificación y Evaluación Sensorial.

[20] Sharapin N., Machado L., Souza E., Rocha E.M., Macedo E.V., Lopes J.M. (2000). Fundamentos de Tecnología de Productos Fitoterapeuticos.

[21] Oliveira A.L., Melo V.L.S., Guimarães A.R., Cabral F.A. (2010). Modelling of high-pressure phase equilibrium in systems of interest in the food engineering field using the peng-robinson equation of state with two different mixing rules. J. Food Process. Eng..

[22] Jokić S., Nagy B., Zeković Z., Vidović S., Bilić M., Velić D., Simándi B. (2012). Effects of supercritical CO_2_ extraction parameters on soybean oil yield. Food Bioprod. Process..

[23] Oliveira N.A., Cornelio-Santiago H.P., Fukumasu H., Oliveira A.L. (2018). Green coffee extracts rich in diterpenes—Process optimization of pressurized liquid extraction using ethanol as solvent. J. Food Process. Eng..

[24] Gião M.S., Pereira C.I., Fonseca S.C., Pintado M.E., Malcata F.X. (2009). Effect of particle size upon the extent of extraction of antioxidant power from the plants *Agrimonia eupatoria*, *Salvia* sp. and Satureja montana. Food Chem..

[25] Burniol-Figols A., Cenian K., Skiadas I.V., Gavala H.N. (2016). Integration of chlorogenic acid recovery and bioethanol production from spent coffee grounds. Biochem. Eng. J..

[26] Farah A., Donangelo C.M. (2006). Phenolic compounds in coffee. Braz. J. Plant Physiol..

[27] De Assis Dias Alves G., De Souza R.O., Rogez H.L.G., Masaki H., Fonseca M.J.V. (2019). *Cecropia obtusa* extract and chlorogenic acid exhibit anti aging effect in human fibroblasts and keratinocytes cells exposed to UV radiation. PLoS ONE.

[28] Zhang Y., Prawang P., Li C., Meng X., Zhao Y., Wang H., Zhang S. (2018). Ultrasonic assisted extraction of artemisinin from: *Artemisia Annua* L. using monoether-based solvents. Green Chem..

[29] Gutiérrez Pulido H., De la vara Salazar R. (2008). Análisis y Diseño de Experimentos.

[30] Karazhiyan H., Razavi S.M.A., Phillips G.O. (2011). Extraction optimization of a hydrocolloid extract from cress seed (*Lepidium sativum*) using response surface methodology. Food Hydrocoll..

[31] Myers R., Montgomery D., Anderson C. (2016). Response Surface Methodology: Process and Product Optimization Using Designed Experiments.

[32] Coelho J.P., Robalo M.P., Boyadzhieva S., Stateva R.P. (2021). Microwave-assisted extraction of phenolic compounds from spent coffee grounds. Process optimization applying design of experiments. Molecules.

[33] Soto-García M., Rosales-Castro M. (2016). Effect of solvent and solvent-to-solid ratio on the phenolic extraction and the antioxidant capacity of extracts from *Pinus durangensis* and *Quercus sideroxyla* bark. Maderas Cienc. y Tecnol..

[34] Manohar M., Joseph J., Selvaraj T., Sivakumar D. (2013). Application of desirability-function and RSM to optimise the multi-objectives while turning Inconel 718 using coated carbide tools. Int. J. Manuf. Technol. Manag..

[35] Castillo-Santos K., Ruiz-López I.I., Rodríguez-Jimenes G.C., Carrillo-Ahumada J., García-Alvarado M.A. (2017). Analysis of mass transfer equations during solid-liquid extraction and its application for vanilla extraction kinetics modeling. J. Food Eng..

[36] Handayani A.D., Sutrisno, Indraswati N., Ismadji S. (2008). Extraction of astaxanthin from giant tiger (*Panaeus monodon*) shrimp waste using palm oil: Studies of extraction kinetics and thermodynamic. Bioresour. Technol..

[37] Lau C., Chua L., Lee C., Aziz R. (2015). Optimization and kinetic modeling of rosmarinic acid extraction from *Orthosiphon stamineus*. Curr. Bioact. Compd..

[38] Parida S., Biswal S. (2020). Kinetics and thermodynamics of lipids extraction from microalgae using n-hexane. Int. J. Energy Appl. Technol..

[39] Butiuk A.P., Maidana S.A., Adachi O., Akakabe Y., Martos M.A., Hours R.A. (2021). Optimization and modeling of the chlorogenic acid extraction from a residue of yerba mate processing. J. Appl. Res. Med. Aromat. Plants.

[40] Lai H.T.N., Nguyen P.V., Tran H.T., Dao V.H.T., Hoang H.H. (2019). Optimization of chlorogenic acid extraction from green coffee beans using response surface methodology. Vietnam J. Agric. Sci..

[41] Dibert K., Cros E., Andrieu J. (1989). Solvent extraction of oil and chlorogenic acid from green coffee. Part II: Kinetic data. J. Food Eng..

[42] Liu Q.M., Yang X.M., Zhang L., Majetich G. (2010). Optimization of ultrasonic-assisted extraction of chlorogenic acid from *Folium eucommiae* and evaluation of its antioxidant activity. J. Med. Plants Res..

[43] Shi J., Yu J., Pohorly J., Young J.C., Bryan M., Wu Y. (2003). Optimization of the extraction of polyphenols from grape seed meal by aqueous ethanol solution. J. Food Agric. Environ..

[44] Dai J., Mumper R.J. (2010). Plant phenolics: Extraction, analysis and their antioxidant and anticancer properties. Molecules.

[45] (1986). Alimentos. Determinación De Humedad En Productos Alimenticios. Foods. Moisture in Food Products Detetermination.

[46] Meinhart A.D., Silveira T.F.F., Silva R.A., Damin F.M., Bruns R.E., Godoy H.T. (2017). Multivariate optimization of chlorogenic acid extraction from Brazilian coffee. Food Anal. Methods.

[47] Ruiz-Palomino P., Guatemala-Morales G., Mondragón-Cortéz P.M., Zúñiga-González E.A., Corona-González R.I., Arriola-Guevara E. (2019). Empirical model of the chlorogenic acid degradation kinetics during coffee roasting in a spouted bed. Rev. Mex. Ing. Quim..

[48] Geankopolis C.J. (1993). Transport Process and Unit Operations.

